# Beyond the iron age: the ecological relevance of non-ferrous bioactive trace metals and organic growth factors in aquatic systems

**DOI:** 10.3389/fmicb.2015.00218

**Published:** 2015-03-20

**Authors:** Laura Gómez-Consarnau, Sergio A. Sañudo-Wilhelmy

**Affiliations:** ^1^Department of Biological Sciences, University of Southern CaliforniaLos Angeles, CA, USA; ^2^Department of Earth Sciences, University of Southern CaliforniaLos Angeles, CA, USA

**Keywords:** vitamin, bacterioplankton, cofactor, vanadium, molybdenum, nitrogen fixation, cobalamin, thiamin

The catalysis of chemical reactions is one of the most central processes in biology, as most reactions in living organisms would occur too slowly to play any role in their metabolism. For example, the rate of biologically relevant reactions would take hundreds to millions of years in the absence of a catalyst (Stockbridge et al., 2010). Enzymes are the catalysts of nature, and they can increase the rate of a chemical reaction by as much as 10^20^ times compared to an uncatalyzed reaction in water (Lad et al., 2003). Molecular evidence suggests that enzymatic catalysis evolved very early in life history, even prior to the Archaean genetic expansion (David and Alm, 2010). While some protein functional groups can act as catalysts, they represent a very limited set of reactions as compared to the multitude of enzyme-catalyzed reactions (Price and Stevens, 1999; Bugg, 2012). Enzymes utilize a variety of non-protein molecules or metal ions to increase their versatility in catalytic capacity. Coenzymes and cofactors are the non-protein components of the enzymes that help catalyze the reactions. Coenzymes are typically vitamin B derivatives while cofactors are molecules or metal ions that are covalently bound to the enzyme (Broderick, 2001).

The multiple oxidation states of some metals increase the functionality of the enzymes. Most of the first row transition metals as well as Mo, W, and Mg are known to function as cofactors in enzymatic catalysis (da Silva and Williams, 2001). Fe is the most abundant transition element in the Earth's crust and, although it is very scarce in the ocean, this element was very abundant at the onset of life (Anbar, 2008). Therefore, it is not surprising that Fe is a major cofactor in some of the most central processes in different biological systems (da Silva and Williams, 2001). Starting with the pioneering work of John Martin and collaborators in the early 1990s (Iron limitation?, 2000; Martin et al., 1991), the so-called *iron hypothesis* has produced major breakthroughs in our understanding of the fundamental role it plays in the marine carbon cycle (Smetacek et al., 2012; Thiele et al., 2012). However, biological activity is not entirely sustained by Fe alone as other elements are used as cofactors in relevant metabolic reactions such as Mo, V, and Ni for N and H_2_ activation respectively, and Cu and Zn in superoxide dismutase (da Silva and Williams, 2001).

B-vitamins are small organic molecules associated with a large number of biologically important enzymes across all domains of life (Sañudo-Wilhelmy et al., 2014). Decades ago, Carlucci, Droop, Provasoli and others showed that the availability of different B vitamins influenced phytoplankton dynamics in the ocean (Droop, 1957; Carlucci and Silbernagel, 1969; Provasoli and Carlucci, 1974; Swift, 1980). Those initial studies also showed that many marine algae require vitamins B_1_(thiamin), B_7_ (biotin), and B_12_ (cobalamin) as growth factors (Provasoli and Carlucci, 1974). Those results have recently been validated as field amendments of different B-vitamins increase phytoplankton growth in many regions of the ocean (Bertrand and Allen, 2012), suggesting that phytoplankton communities in large areas are indeed limited by the availability of these organic metabolites. The initial notion that B-vitamin auxotrophy (i.e., B-vitamin requirement) only applies to phytoplankton species has been reevaluated as it is now well accepted that some bacterial taxa are also auxotrophic (Giovannoni et al., 2005; Croft et al., 2006; Carini et al., 2014; Sañudo-Wilhelmy et al., 2014). Therefore, both vitamin-producing and -consuming bacteria and algae seem to coexist in the ocean. However, more studies are needed to understand the ecological role of the B-vitamins in the marine environment.

A total of 10 manuscripts are included in this e-book being published in Frontiers in Microbiology; 7 are original research (3 on vitamins and 4 on trace metals), 2 reviews and 1 methods article that address some aspect of bioactive trace elements other than Fe, as well as B-vitamins in aquatic environments. The two review articles published focused on the transition element Mo. Glass et al. (2012) reviewed the current state of knowledge regarding Mo limitation in aquatic systems (Glass et al., 2012). One of their major conclusions is that Mo availability seems to influence the N cycle in some freshwater and soil environments, although the authors also concluded that the impact of this element in open ocean waters have not been thoroughly studied. The mini-review by Wang (2012) seeks to elucidate a connection between the evolution of different Mo-requiring enzymes with historical changes in the redox chemistry of Mo in the ocean (Wang, 2012). Those two reviews are complemented by the work of Romero et al. (2013) showing that, in some lakes with different trophic status, field amendments of a specific chemical form of Mo enhanced nitrogen fixation and biomass concentrations (Romero et al., 2013). The biological role of Mo and its geochemical analog, V, in open ocean waters was evaluated in the article of Klein et al. (2013). They found statistical correlations between intracellular levels of Mo measured in natural phytoplankton communities and dimethyl sulfide (DMS) concentrations as well as between intracellular V concentrations and chlorophyll a in the North East Atlantic. While the authors stated that the Mo-DMS relationship was not unexpected as the synthesis of DMS is catalyzed by the Mo-containing enzyme dimethyl sulfoxide reductase, they did not put forward an explanation for the biological role of V in their study. Indeed, V availability could enhance chlorophyll-a biosynthesis by increasing the synthesis of the chlorophyll-precursor, 5-aminolevulinic acid, via the C5 pathway as has previously been observed (Meisch and Bielig, 1975). However, further studies are needed to test the V-chlorophyll connection. The biological importance of another under-studied transition element, Ni, is explored in the article of Ho et al. (2013) who studied the effect of light intensity and Ni availability on *Trichodesmium* growth. The authors concluded that high intracellular levels of Ni in the diazotroph measured under illumination were probably associated with the activity of the Ni-containing superoxide dismutase enzyme that catalyzes the disproportionation of the superoxide radical formed during photosynthesis. The article by Mackey et al. (2012) explores how the atmospheric deposition of different metals could selectively affect phytoplankton community dynamics in coastal and open ocean waters of the Sargasso Sea. Their work showed that the biological response to the aerosol-derived transition trace metal additions was spatially dependent, as the greater biological drawdown of dissolved Co, Mn, and Ni occurred in the open ocean. Therefore, although aerosol metal additions did not cause a shift in the phytoplankton communities, the growth responses were different across ocean regimes. Mackey et al. (2012) hypothesized that the varying biological response was dependent on the metal-nutritional status of the resident phytoplankton prior to the aerosols input, although establishing background metal concentrations prior to addition experiments has faced analytical difficulties due to the complexity of metal analyses at the levels found in open ocean waters. The article of Durand et al. (2012) is a step-forward in the direction to solve that problem. They developed a new microplate-reader method for the rapid analysis of Cu in natural waters that requires less than 1 milliliter of sample and can process about 100 samples per hour. Although Durand et al. (2012) recognized that the analytical method has to be optimized prior to use in open ocean waters, they provide a series of recommendations that could increase the usefulness of their technique. Three articles published in this ebook concentrated on B-vitamins in different marine environments. The articles of Barada et al. (2013) and Bonnet et al. (2013) established spatial gradients in the Mediterranean Sea and in the Amazon River plume respectively. These two studies significantly increased the geographical database of ambient B-vitamin concentrations in the ocean. As observed in other regions (Sañudo-Wilhelmy et al., 2012), those two studies also showed that large areas in Mediterranean and in the Western Tropical North Atlantic are devoid of vitamins. However, Barada et al. (2013) and Bonnet et al. (2013) found significant correlations between different vitamin levels and chlorophyll a concentrations as well as with C and N fixation respectively at some locations, suggesting a potential role of those coenzymes on ecosystem dynamics. The effect of vitamins B_1_ and B_12_ on coastal ecosystem dynamics was the main objective of the article of Koch et al. (2012). The authors established the effect of vitamin additions on the planktonic community composition, carbon fixation, and B-vitamin assimilation in two costal sites off Long Island, New York with different trophic characteristics. The reported results were counterintuitive, as dissolved vitamin concentrations and uptake rates were higher in the most eutrophic environment. Koch et al. (2012) concluded that the major B-vitamin consumers in their study sites were heterotrophic bacteria, consistent with genomic results indicating that some heterotrophs are B-vitamin auxotrophs (Sañudo-Wilhelmy et al., 2014). In summary the articles published in this ebook represent a significant advance in our understanding of the roles that trace metals other than Fe and B-vitamins play in the marine environment.

## Conflict of interest statement

The authors declare that the research was conducted in the absence of any commercial or financial relationships that could be construed as a potential conflict of interest.
